# A rare entity of reactive arthritis‐immune reconstitution inflammatory syndrome in an immunocompromised patient

**DOI:** 10.1002/ccr3.2258

**Published:** 2019-06-23

**Authors:** Christine Katusiime

**Affiliations:** ^1^ Infectious Diseases Institute College of Health Sciences, Makerere University Kampala Uganda; ^2^ Ministry of Health Kampala Uganda

**Keywords:** antiretroviral therapy, human immunodeficiency virus, immune reconstitution inflammatory syndrome, reactive arthritis

## Abstract

There is increasing association of reactive arthritis (ReA) with the human immunodeficiency virus (HIV). ReA‐immune reconstitution inflammatory syndrome (IRIS) on the contrary is such a rare occurrence that it has only been documented twice. This is the index documented case of ReA‐IRIS in sub‐Saharan Africa.

## 1 INTRODUCTION

Reactive arthritis (ReA) is a seronegative spondyloarthropathy characterized by articular and extra‐articular manifestations. The classical triad of the syndrome illustrated by arthritis, urethritis, and conjunctivitis, however, is only observed in a third of patients.^1^, ^2^ Because the diagnosis of ReA is rather problematic, owing to the fact that identification of the triggering bacterium is relatively difficult at the time when arthritis occurs, two sets of diagnostic criteria have been established by the American College of Rheumatology (ACR) and the third international workshop on reactive arthritis to this effect. 3We describe a rare case of ReA‐immune reconstitution inflammatory syndrome with debilitating articular and extra‐articular manifestations.

## 2 CLINICAL REPORT

A 40‐year‐old HIV‐positive man first presented to the Infectious Diseases Institute, Kampala, with World Health Organization (WHO) stage III disease having had a history of recurrent low‐grade fevers for more than a month. His index clinical visit showed that he had a body weight of 55 kg and a baseline CD4+ count of 61 cells/µL (7%). The patient was lost to follow up for about 9 months and was thereafter actively traced and incorporated back into care.

The patient presented on the next clinic visit, which was 9 months after the index visit, with a 2‐week history of dysuria, frequency, and low‐grade fevers which was managed as nongonococcal urethritis (NGU) following urinalysis with a 10‐day course of ciprofloxacin and nonsteroidal anti‐inflammatory drugs (NSAIDs). There was no associated history of frequency, urgency, burning micturition, hematuria, or abnormal penile discharge. General and genital‐urinary examinations were unremarkable. Urine cultures showed no growth.

Four weeks later, the patient presented with a 1‐week history of frequency, urgency, burning micturition, urinary retention, and oral sores which were managed as NGU, prostatitis, and herpetic stomatitis with a 14‐day and 7‐day course of ciprofloxacin and acyclovir, respectively. Physical examination also revealed hypo‐pigmented skin lesions on the upper arms which was diagnosed as pityriasis versicolor and managed with clotrimazole cream.

Three weeks following this, he presented with a 1‐week history of painful penile sores, yellowish non–foul‐smelling penile pus discharge, bilateral conjunctivitis, and mild bilateral knee joint pains which was managed as gonococcal urethritis and allergic conjunctivitis with a 2‐week course of ciprofloxacin, NSAIDs, and hydrocortisone eye drops, respectively. Fundoscopy was unremarkable.

On this visit, he was concurrently initiated on highly active antiretroviral therapy (HAART)—zidovudine, lamivudine, and nevirapine of which he was fully compliant. As a consequence, there was remarkable CD4+ cell count increase to 450 cells/µL. Subsequent monthly clinic visits were characterized by complaints of worsening joint pains which were generally managed with short courses of NSAIDs.

Exactly 23 weeks (5 months) following HAART initiation, he presented with such debilitating bilateral knee joint pains that there was associated severe movement restrictions, joint swelling, and joint stiffness.

Physical examination revealed scattered maculopapular skin rashes on the chest and upper back. Musculoskeletal examination revealed bilateral tender knee joints’ swelling with effusion and joint movement restrictions anteriorly, posteriorly, and laterally. Fundoscopy was unremarkable. Gastrointestinal and genital examinations were essentially normal, and the rest of the systemic examination was unremarkable. Complete blood counts (CBC) revealed an elevated erythrocyte sedimentation rate (ESR) of 100 mm/h (0‐15 mm/h). Other biochemistry tests including serum electrolytes, liver, and renal function tests were unremarkable.

Treponemal pallidum hemagglutination assay (TPHA) was nonreactive. Rheumatoid factor, serum uric acid, HBsAg, and hepatitis C antigen assays were negative. Knee joint X‐rays showed soft tissue swelling. Synovial fluid aspiration showed a leucocytosis on cytology with no bacterial growth on cultures. Antinuclear antibody tests and HLA‐B27 testing could not be done because of the high costs involved.

At this point, a comprehensive review of his past clinical history demonstrated that the combination of urethritis, arthritis, and conjunctivitis completed the triad of reactive arthritis. At that point in time, the clinical diagnosis of reactive syndrome immune reconstitution inflammatory syndrome was made. His CD4+ counts and viral load were 450 cells/µL and 0 copies/mL, respectively.

The patient was encouraged to continue HAART and was commenced on steroid therapy—prednisone 40 mg/d which was tapered off gradually over a month, NSAIDs and a weak opioid. There was remarkable improvement in the patient's condition, and after 1‐month treatment, he resumed his daily activities.

## 3 DISCUSSION

Immune reconstitution inflammatory syndrome (IRIS) is still a serious and potentially fatal complication of HAART initiation. The incidence of IRIS is more severe in developing countries because of the co‐existence of high degrees of immune‐suppression and high prevalence of opportunistic infections.^3^

HIV/AIDS is associated with frequent articular manifestations that are often underdiagnosed. Articular manifestations in HIV may range from HIV‐associated arthralgias, reactive arthritis, psoriatic arthritis, painful articular syndrome, spondylarthritis, rheumatoid arthritis, systemic lupus erythematosus, diffuse infiltrative lymphocytosis syndrome (DILS; Sjogren's‐like syndrome), septic arthritis, vasculitis, myopathy, undifferentiated spondyloarthropathies, avascular necrosis, fibromyalgia, hypertrophic pulmonary osteoarthropathy, osteoporosis to osteopenia.^4^, ^5^, ^6^

The clinical manifestation of reactive arthritis generally consists of six categories: urogenital, rheumatologic, ophthalmologic, dermatologic, oral, and visceral.^7^ This is the first documented observation of reactive arthritis‐IRIS in sub‐Saharan Africa and the first on the African continent. Literature review, however, revealed only two previous cases described in Germany and India.^1^, ^2^

Our patient exhibited the triad of urogenital, rheumatologic, and ophthalmologic criteria which is distinctive of reactive arthritis (arthritis, conjunctivitis, and urethritis).

Reactive arthritis in HIV is generally rare. This is attributed to the fact that the use of prophylactic measures like co‐trimoxazole has been demonstrated to confer a protective effect against genitourinary and enteric infections which have been implicated in the pathogenesis of reactive arthritis.^8^

Although HLA‐B27 testing was not done in our patient due to the high costs involved, it has been found to be associated with HIV‐associated reactive arthritis in 80%‐90% of Caucasians. Studies in Africans on the contrary have shown that the majority of Africans are HLA‐B27 negative.^8^, ^9^ HLA‐B27 positivity in patients has not only been found to be associated with an increased probability of developing various extra‐articular manifestations but is also associated with an increased severity of reactive arthritis.^9^, ^10^

An antecedent history of genitourinary infection prior to HAART initiation is what predisposed our patient to reactive arthritis IRIS.

Although there is an increased probability of HIV‐positive persons with reactive arthritis developing extra‐articular manifestations for instance plantar fasciitis, dactylitis, and achilles tendonitis,^10^, ^11^ our patient did not develop any extra‐articular feature.

This case also highlights a key challenge in providing HAART to patients in resource‐limited settings; the fact that the majority of HIV‐infected persons access HIV care with advanced immunosuppression and low CD4 counts.

Results from a randomized controlled trial in South Africa demonstrated that use of prophylactic prednisone during the first 4 weeks after HAART initiation resulted in a 30% reduction in incidence of tuberculosis‐associated IRIS in patients at high risk for tuberculosis‐associated IRIS.^11^, ^12^

There is a possibility that IRIS would not have progressed in the pattern it did, had the patient been provided with prophylactic prednisone in the first month of HAART initiation. Because rheumatologic manifestations are common in HIV, future studies could evaluate the degree of articular manifestations in HIV‐infected persons in the presence of a prophylactic steroid during HAART initiation.

## CONFLICT OF INTEREST

None declared.

## AUTHOR CONTRIBUTION

CK: is the corresponding author; clinician; involved in the study, design, and analysis of data; drafted and critically revised the manuscript.

## CONSENT FOR PUBLICATION

Not applicable (we have not included names, images, or videos that need obtaining of consent).
